# Malignant perivascular epithelioid cell neoplasm in the liver: report of a pediatric case

**DOI:** 10.1186/s40792-021-01300-w

**Published:** 2021-09-20

**Authors:** Tokuro Baba, Takafumi Kawano, Yusuke Saito, Shun Onishi, Koji Yamada, Waka Yamada, Ryuta Masuya, Kazuhiko Nakame, Yota Kawasaki, Satoshi Iino, Masahiko Sakoda, Mari Kirishima, Tatsuru Kaji, Akihide Tanimoto, Shoji Natsugoe, Takao Ohtsuka, Hiroshi Moritake, Satoshi Ieiri

**Affiliations:** 1 Department of Pediatric Surgery, Research Field in Medicine and Health Sciences, Medical and Dental Sciences Area, Research and Education Assembly, Kagoshima University, Kagoshima, Japan; 2 Division of Pediatrics, Faculty of Medicine, University of Miyazaki, Miyazaki, Japan; 3 Department of Digestive Surgery, Breast and Thyroid Surgery, Research Field in Medicine and Health Sciences, Medical and Dental Sciences Area, Research and Education Assembly, Kagoshima University, Kagoshima, Japan; 4 Division of the Gastrointestinal, Endocrine and Pediatric Surgery, Department of Surgery, University of Miyazaki Faculty of Medicine, Miyazaki, Japan; 5 Department of Pathology, Research Field in Medicine and Health Sciences, Medical and Dental Sciences Area, Research and Education Assembly, Kagoshima University, Kagoshima, Japan

**Keywords:** Perivascular epithelioid cell neoplasm (PEComa), ^18^F-fluorodeoxyglucose-positron emission tomography (FDG-PET), Hepatocellular carcinoma, Segmentectomy

## Abstract

**Background:**

Perivascular epithelioid cell neoplasm (PEComa) in a child is very rare. We herein report the first malignant case of PEComa developing in the liver of a pediatric patient.

**Case presentation:**

A 10-year-old boy visited a private clinic with prolonged fever of unknown etiology. Abdominal ultrasonography was performed to evaluate the fever’s origin, revealing a large tumor in the liver. He was thus referred to a nearby hospital to investigate the tumor further. Enhanced computed tomography (CT) showed a 6.8 × 5.9 × 10.5-cm solid lesion on S4 and S5. On magnetic resonance imaging (MRI), the tumor had a low signal intensity on T1 imaging and high signal intensity on T2 imaging, with partial diffusion restriction. ^18^F-fluorodeoxyglucose-positron emission tomography (FDG-PET) showed a marked uptake in the mass lesion with no evidence of metastasis. The patient was negative for all tumor markers, including AFP, CEA and PIVKA-II. The results of a needle biopsy suggested hepatocellular carcinoma. The tumor’s rapid growth suggested malignancy. Hepatic segmentectomy (S4 + S5 + S8) was performed. The tumor was resected en bloc with a margin. Microscopically, the tumor showed atypical spindle, polygonal or oval-shaped cells with a high nuclear grade, and vascular invasion. Immunohistochemistry was positive for alpha-smooth muscle antigen (α-SMA), human melanin black-45 (HMB-45) and melan A. The pathological diagnosis was malignant PEComa. In the 6 months after surgery, the patient complained of shoulder pain. MRI showed a dumbbell-shaped tumor at the 2nd thoracic vertebrae, which was confirmed to be bone metastasis of PEComa. After chemotherapy, including ifosfamide and doxorubicin, vertebrectomy was performed. Two years later, thoracoabdominal CT showed a 10-cm solid mass occupying the pelvis and a 15-mm nodule in the middle lobe of the right lung. Under a diagnosis of peritoneal and lung metastases, they were surgically removed and metastasis of PEComa was pathologically confirmed. Four months after the 2nd relapse, pelvic metastasis appeared again and mTOR (mammalian target of rapamycin) inhibitor was initiated. To our knowledge, this is the first report of malignant hepatic PEComa in a pediatric patient.

**Conclusion:**

Although extremely rare, malignant hepatic PEComa can develop in a child.

## Background

The term perivascular epithelioid cell neoplasm (PEComa) was first described by Zamboni et al. in 1996 [1] to represent a group of mesenchyme-derived neoplasms primarily composed of histologically distinctive perivascular epithelioid cells [2]. This group of tumors is now called the PEC tumor family and includes angiomyolipoma (AML), clear-cell sugar tumor of lung (CCST) and lymphangioleiomyomatosis (LAM), along with a number of immunophenotypically and morphologically similar rare tumors arising from the abdominal organs, soft tissues and bones [3].

However, the definitions of PEComa and AML have often been confused. Strictly, the term ‘PEComa’ is reserved for the group of tumors and is also used to refer to relevant lesions that cannot be specifically classified as AML, CCST or LAM [4]. However, these lesions are regarded by many global experts as the same entity. The incidence of PEComa is not clear, even in adult patients, but it is known to be very rare, especially in children. The biological characteristics of PEComa vary widely, ranging from benign to malignant. Most cases of PEComa have been described as showing benign behavior, but a few malignant cases in children have been reported in the literature [3, 5]. Still, such lesions are quite rare, with fewer than 40 malignant PEComas having been reported in pediatric populations around the world [6].

To our knowledge, there have been no previous reports of a malignant hepatic PEComa developing in a child. Therefore, we report the first pediatric case of a malignant PEComa that developed in the liver of a 10-year-old boy.

## Case presentation

A 10-year-old boy who was otherwise healthy visited a private practice due to high fever, which had persisted for about 1 month. A blood test showed a high level of C-reactive protein (13.1 mg/dl; normal value: < 0.01 mg/dl), so he was referred to a nearby hospital for a further investigation.

**Fig. 1:**
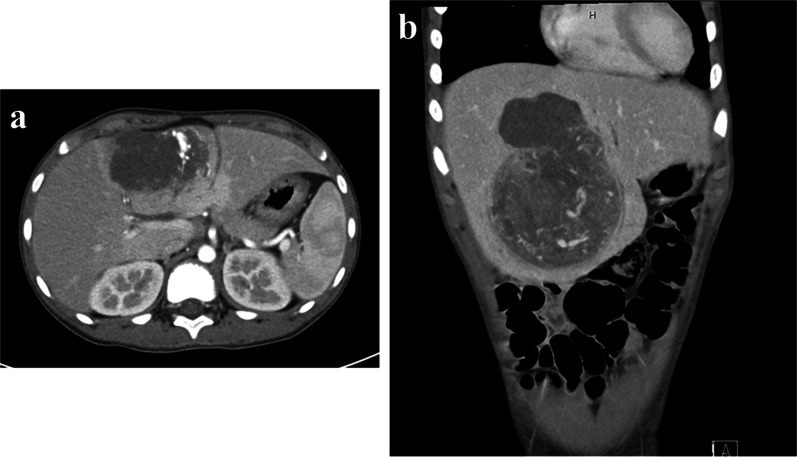
CT findings. CT shows a 7.7 × 8.8 × 12.0-cm tumor located mainly in S4 and S5 of the liver (**a**). It was well-circumscribed and showed gradual weak enhancement in both the arterial and portal phases (**b**)

**Fig. 2:**
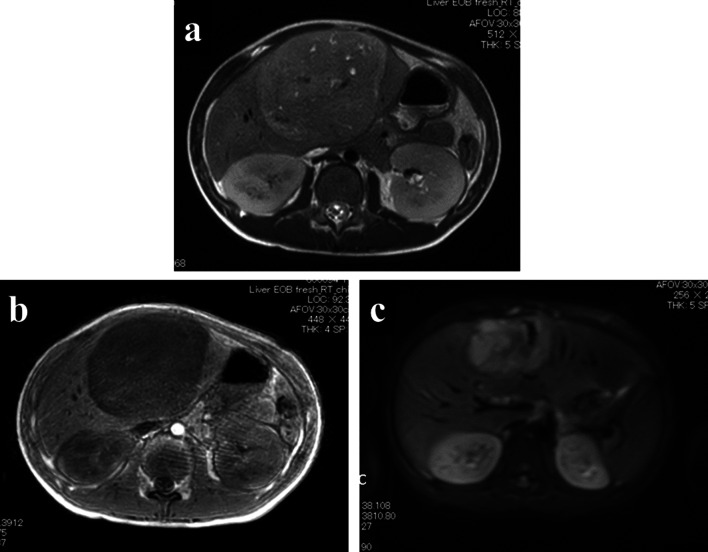
MRI findings. The tumor shows low intensity on T1-weighted imaging (**a**) and high intensity on T2-weighted imaging (**b**) with partial diffusion restriction (**c**)

**Fig. 3:**
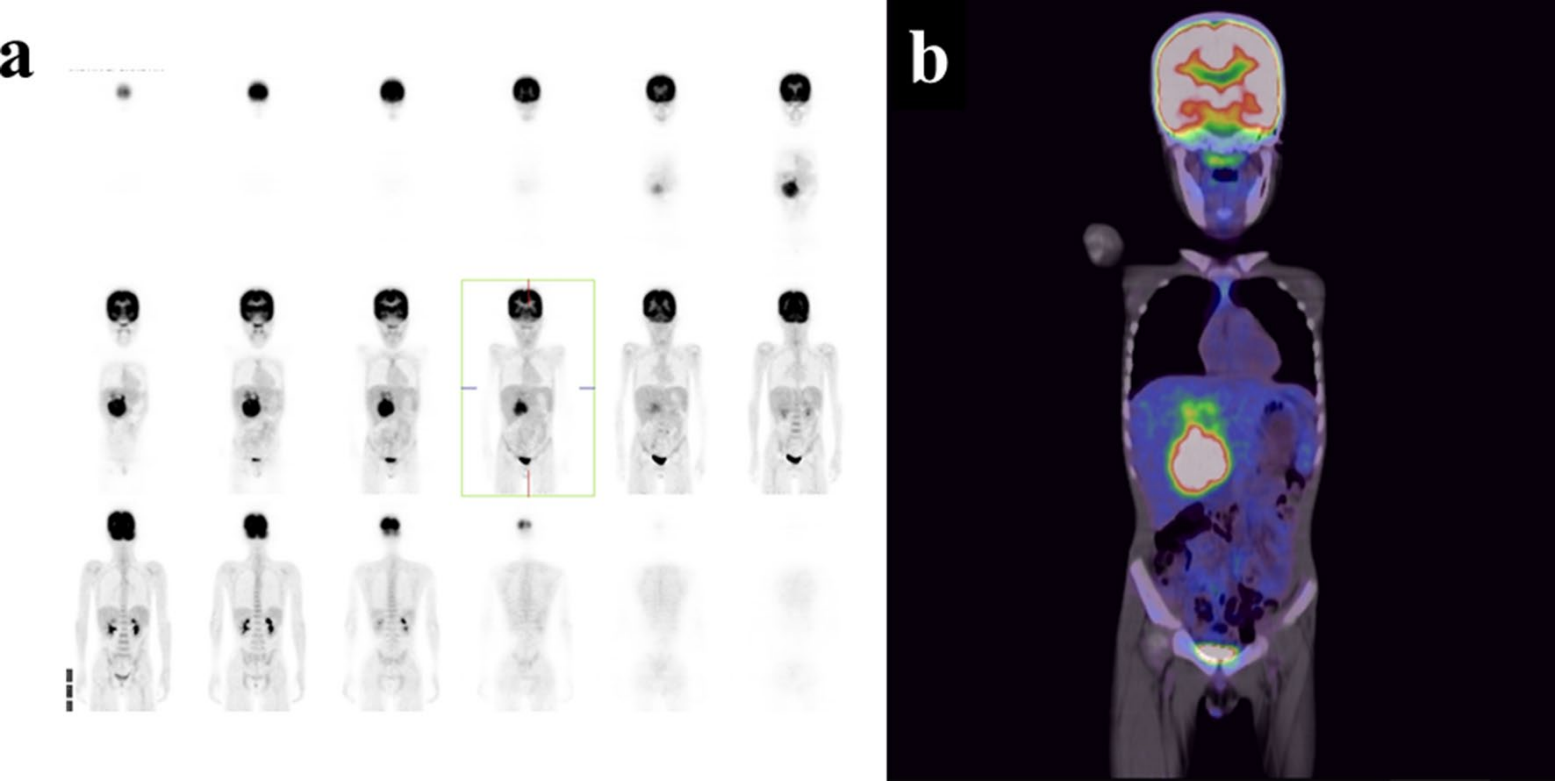
FDG-PET findings. FDG-PET shows the tumor to have a high FDG uptake (SUVmax: 8.8) (**b**) with no signs of distant metastasis (**a**)

Abdominal ultrasonography at that hospital revealed a solid large tumor in the liver (approximately 6.3 × 5.3 cm at the time). Enhanced computed tomography (CT) showed that the tumor was mainly located at S4 and S5 in the liver (Fig. 1). The tumor was well-circumscribed and showed gradual weak enhancement from the arterial phase to the portal phase. On magnetic resonance imaging (MRI), the tumor showed low intensity on T1-weighted imaging and a high intensity on T2-weighted imaging, with partial diffusion restriction (Fig. 2). ^18^F-fluorodeoxyglucose-positron emission tomography (FDG-PET) showed the marked uptake of FDG by the tumor, with an early maximum standardized uptake value (SUV_max_) of 8.8 (Fig. 3). There was no evidence of distant metastasis on any of the imaging modalities.

The levels of tumor markers, including α-fetoprotein (AFP), protein induced by vitamin-K antagonist-II (PIVKA-II), carcinoembryonic antigen (CEA), and carbohydrate antigen 19-9 (CA19-9), were all within the normal limits. Based on these findings, the most likely differential diagnosis was suspected to be undifferentiated sarcoma; thus, he was referred to our hospital for surgical treatment.

**Fig. 4:**
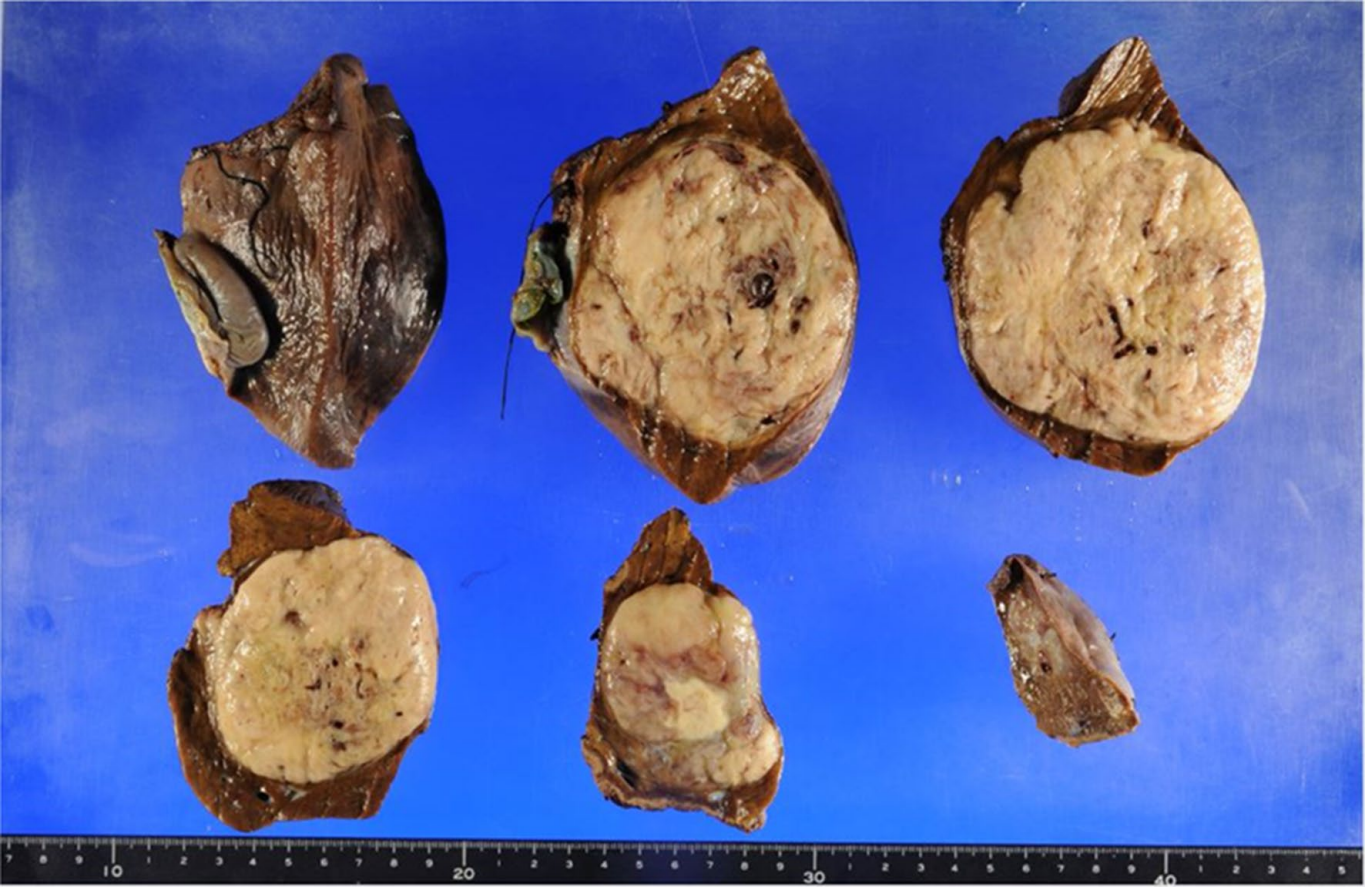
Histopathological findings (macroscopic). A yellowish white, solid 10 × 9-cm tumor is seen, showing hemorrhaging and necrosis

**Fig. 5:**
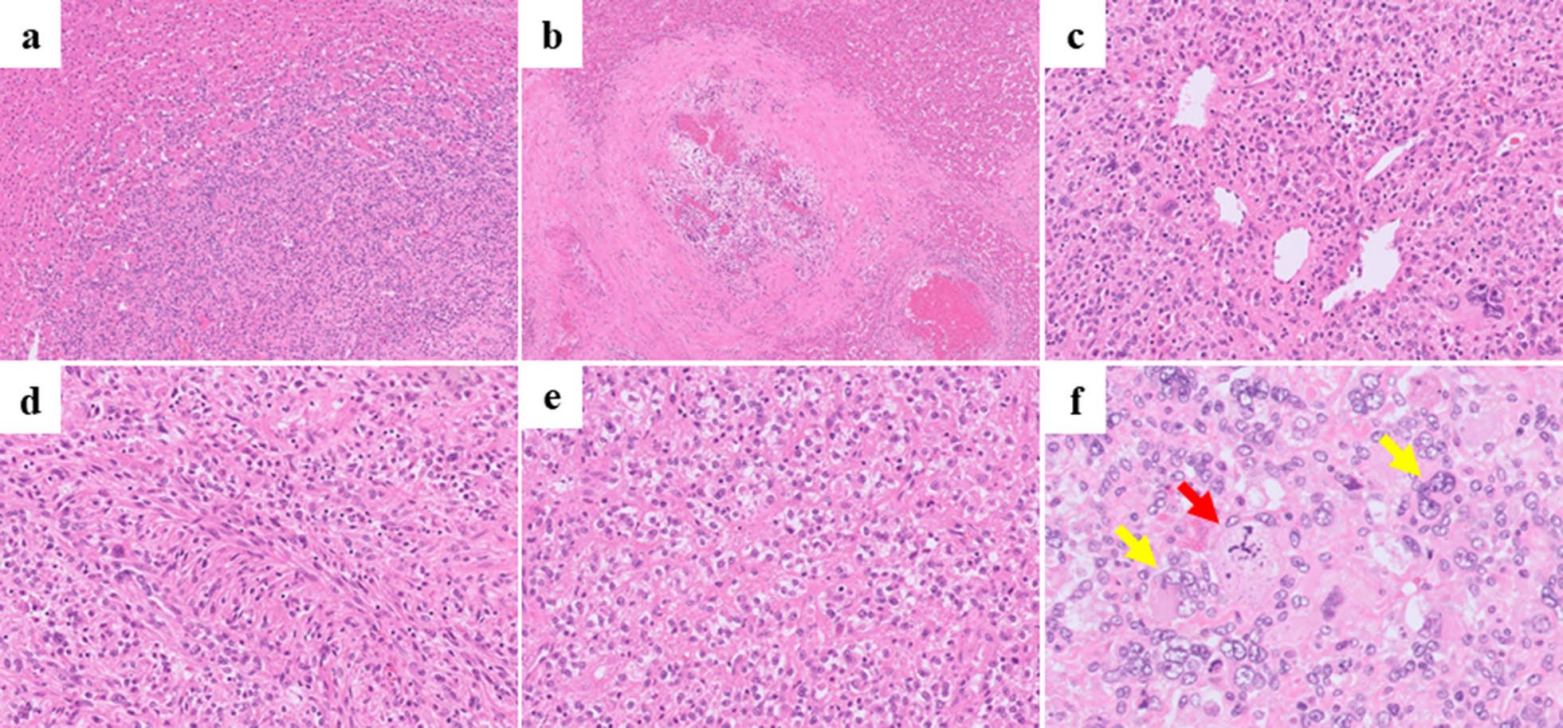
Histopathological findings (microscopic). The tumor shows an infiltrative growth pattern (**a**) and vascular invasion is also noted (**b**). The tumor is composed of polygonal or oval-shaped cells arranged around blood vessels (**c**), spindle cells arranged in fascicles (**d**), and round epithelioid cells with clear cytoplasm (**e**). Multinucleated giant cells are also noted (**f**, highlighted with yellow arrows). Mitotic figure was easily recognized, including abnormal mitosis (**f**, highlighted with a red arrow)

**Fig. 6:**
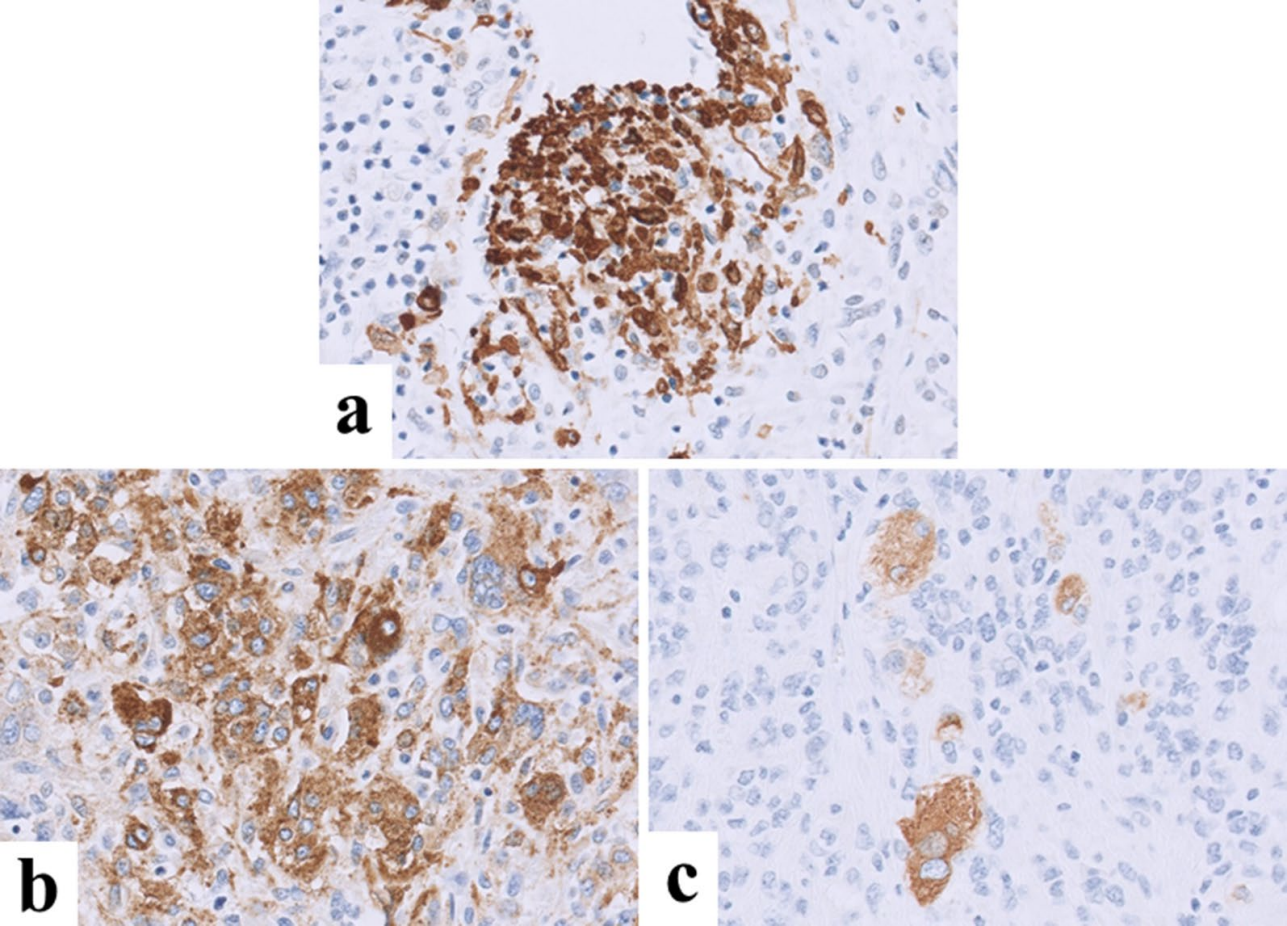
Immunohistochemical findings. Immunohistochemistry showed α-SMA (**a**) and melan A (**c**) were positive in some cells, while HMB-45 (**b**) was positive in about 50% of cells

First, to confirm the diagnosis and develop an operation plan, a core needle biopsy was performed at our hospital. Based on the findings of the preoperative examination, including the biopsy results, the preoperative diagnosis was suspected hepatocellular carcinoma. The Child–Pugh score was 8 (albumin, 3; prothrombin time, 2; bilirubin, 1; ascites, 1; and encephalopathy, 1), and the indocyanine green retention rate (ICG-R15) was 2.9%. On enhanced CT just before the operation, the tumor was 8.7 × 10.4 × 13.1 cm in size, showing marked growth within a one-month period. We planned to perform elective surgical resection by laparotomy. At the operation, a large mass was located in the central portion of liver with no findings of peritoneal dissemination or intra-abdominal metastasis. The tumor involved liver segments 4, 5 and 8; however, the dorsal portion of segment 8 had been spared. The tumor did not actually invade Glisson’s sheath at the liver hilum, and the distance between the tumor and the umbilical portion of portal vein was 1 cm. After removing the gallbladder, hepatic parenchymal transection was performed with a water-jet hybrid knife (erbe JET2^®^; Erbe Elektromedizin GmbH, Tubingen, Germany) using the Pringle maneuver while confirming the tumor location using intraoperative ultrasonography. The dorsal portion of Segment 8 was successfully preserved. The blood supply of the area was confirmed by intraoperative ultrasonography after resection. Finally, the tumor was resected en bloc with a margin. The operative time was 521 min, and the blood loss was 490 ml. He was transferred to the previous hospital on postoperative day 12. Macroscopic findings showed a yellowish white, solid tumor of 10 × 9 cm in size with hemorrhage and necrosis (Fig. 4). Although the tumor was well-circumscribed macroscopically, in a histopathological examination, the tumor cells showed an infiltrative growth pattern (Fig. 5a) and vascular invasion was observed (Fig. 5b). The tumor was composed of polygonal or oval-shaped cells arranged around blood vessels (Fig. 5c), spindle cells arranged in fascicles (Fig. 5d), and round epithelioid cells with clear cytoplasm (Fig. 5e). Tumor cells showed a high nuclear grade and multinucleated giant cells were also noted (Fig. 5f, indicated with yellow arrows). Mitotic figures were easily recognized; the mitotic activity was 30/50 hpf, including abnormal mitosis (Fig. 5f, highlighted with a red arrow). Immunohistochemistry revealed that some cells were positive for α-SMA and melan A, while approximately 50% of cells were positive for HMB-45 (Fig. 6). These histopathological findings, along with immunoreactivity with melanocytic markers, were consistent with a diagnosis of perivascular epithelioid cell tumor. The diagnosis was also confirmed by a central review committee in the Japan Children’s Cancer Group (JCCG). After discharge from our hospital, he was followed up at another hospital. In the 6th month after the initial surgery, he complained of shoulder pain. MRI showed dumbbell-like shaped tumor at the 2nd thoracic vertebrae, which was confirmed to be bone metastasis of PEComa by biopsy. After reducing the tumor size by chemotherapy (including ifosfamide and doxorubicin), vertebrectomy was performed. Postoperatively he suffered from cerebrospinal fluid leakage and meningitis, which were treated by vancomycin. Additional therapy was judged to be unnecessary because no viable cells were found in the specimen. At two years from the relapse, in a regular follow-up visit, thoracoabdominal CT showed a 10-cm solid mass occupying the pelvis and a 15-mm nodule in the middle lobe of the right lung. The pelvic tumor was extirpated by laparotomy, while a nodule in right lung was removed in a thoracoscopic procedure. A pathological examination revealed that both lesions were PEComa, and genetic alteration of the TSC2 gene was identified in tumor cells. At four months after second relapse, pelvic metastasis appeared again. Since the third relapse, he has been carefully treated with a mammalian target of rapamycin (mTOR) inhibitor.

## Discussion

PEComa is a mesenchymal neoplasm with no definite origin that mainly affects young female adults [7]. Pediatric cases are rare. Thus far, fewer than 40 PEComas in children have been reported in the relevant literature [6]. PEComa can occur anywhere, including the uterus, vulva, rectum, urinary bladder, abdominal wall and pancreas [8, 9]. However, hepatic PEComa is relatively rare. Maebayashi et al. conducted a systematic review of hepatic PEComas [10], reporting nine men and 64 women with a median age of 46 years old and minimum age of 17 years among both PEComa and AML patients. PEComa seems to be an extremely rare entity among pediatric liver tumors. Furthermore, the biological behavior of most PEComas, despite being able to occur almost anywhere, is largely benign, with only a small portion showing malignant behavior [3, 5]. Even in adults, the number of case reports of malignant hepatic PEComa are limited (Table 1). To our knowledge, this is the first report to describe the development of malignant hepatic PEComa in a child.

**Table 1:** Review of 18 cases of malignant hepatic PEComa in adults reported in the literature

Author	Year	Sex/age	Size (cm)	Location	CT finding	MRI finding	Surgical procedure	Recurrence site	Chemotherapy	Prognosis
Dalle et al. [28]	2000	F/70	15.0	Right lobe	Hypervascular	NP	Right trisegmentectomy	Liver	NP	7 months alive from surgery
Flemming et al. [29]	2000	F/51	10 and 0.5	left lobe	NA	NA	Left lobectomy → Right lobectomy	Liver	NP	1.5 years alive without disease
Mizuguchi et al. [30]	2004	F/49	NA	Right lobe	Hypervascular	NA	Right trisegmentectomy	No	NP	NA
Parfitt et al. [5]	2006	F/60	14.0	Right lobe	NA	NP	Right lobectomy	Liver, trapezius, bladder, lung, and pancreas	NP	NA
Yang et al. [31]	2007	F/37	13	Left lobe	NA	NA	Extended left lobectomy	Liver, lung	NA	Died 14 months after surgery
Deng et al. [32]	2008	F/30	11	Right lobe	Hypervascular	NP	Right lobectomy	Liver, pancreas, and lung	Adjuvant	Died 4 months after recurrence
Nguyen et al. [27]	2008	F/43	11	Left lobe	Na	NA	Left hepatectomy	Liver, peritoneum, retroperitoneum, and omentum	NP	Died 3 months after surgery
Kamimura et al. [33]	2010	F/43	11.0	Left lobe	Early enhancement, wash-out in delayed phase	Low on T1I, High on T2I	Left lobectomy	No	NP	Disease free in 3 years
Selvaggi et al. [34]	2011	F/68	9.0	Right lobe	Hypodense lesion, multiple lesions around triangular ligament, hemoperitoneum	NP	Omentectomy, toilette, hemostasis	No	NP	Died 25 days after admission
Ding et al. [35]	2011	F/31	NA	Right lobe	NA	NA	Right lobectomy	Liver	NP	Died 1 year after surgery
Kobayashi et al. [36]	2013	F/60	NA	Right lobe	Hypodense lesion, persistent enhancement	Low on T1I High on T2I High on DWI	Partial hepatectomy	No	NP	Disease free in 3 years
Bergamo et al. [23]	2014	M/30	18.0	Right lobe	NP	Hypervascular	Partial hepatectomy	No	Sirolimus	alive
Abhirup et al. [4]	2015	F/49	10	Right lobe	Heterogeneously enhanced with central necrosis	No	Extended right hepatectomy	Liver	NP	Died 12 months after recurrence
Wang et al. [20]	2015	F/37	9	Left lobe	Early enhancement, wash-out in delayed phase	Low on T1I High on T2I High on DWI	Hepatectomy → Liver transplantation	Liver	NP	Alive
Hao et al. [18]	2016	F/51	8.0	Right lobe	Hypodense lesion, brightly enhance, slightly local hyperattenuating	NP	Hepatectomy	NA	NP	Alive
Fukuda et al. [37]	2016	M/58	6.3	Right lobe	Early enhancement, wash-out in delayed phase	NP	Anterior segmentectomy → Partial pneumonectomy	Lung	NP	Recurrence free in 2 years from surgery
Liu et al. [38]	2016	M/34	15	Left lobe	Early enhancement, wash-out in delayed phase	NA	Left lobectomy	No	NP	Alive
Liu et al. [38]	2016	M/31	3	Right lobe	Early enhancement, wash-out in delayed phase	NA	Right lobectomy	No	NP	Alive
Our case	2021	M/10	13.1	Central portion	Weakly enhanced in the both arterial and portal phase	Low on T1I High on T2I Relatively high on DWI	Central bisegmentectomy	Thoracic vertebrae, lung and pelvis	Ifosfamide, doxorubicin/everolimus	Alive

Eighteen cases were identified. Female predominance is obvious, and right lobe was more often affected. Surgical procedures most often performed were hepatic lobectomy, followed by segmentectomy, and partial hepatectomy. CT was performed for most patients, but MRI was limited in number. mTOR inhibitor was administered as neoadjuvant therapy to only one patient. *F* female, *M* male, *NA* information not available, *NP* not performed, *T1I* T1-weighted imaging, *T2I* T2-weighted imaging, *DWI* diffusion-weighted imaging

The preoperative definitive diagnosis of PEComa based on radiology is considered very difficult. Chen et al. reported the imaging characteristics of seven patients with hepatic PEComa [11]. The CT images of four patients who underwent enhanced scanning showed that the lesion became intensely enhanced in three patients (75%), with no clear enhancement observed in one patient (25%). The images from the portal and delayed phases showed variable findings. In the present case, the tumor gradually showed weak enhancement in both the arterial and portal phases. CT findings are not useful for differentiating PEComa. In the study by Chen et al. on MRI, 3 cases showed a slightly hypointense signal on T1-weighted imaging and a slightly hyperintense signal on T2-weighted imaging (100%) [11]. These results are compatible with those of our case. Since these findings are not typical of PEComa, MRI findings alone cannot lead to a definitive diagnosis of PEComa.

A definitive diagnosis of PEComa can only be obtained by pathological examinations. Fine-needle aspiration biopsy (FNAB) has been considered mandatory for a majority of patients [12]; however, a definite diagnosis was achieved in only a few cases [13]. This may be because the tissue volume obtained by FNAB is not sufficient to make a diagnosis, and PEComas are not often considered by pathologists due to their rarity, especially in children. In our case, we first suspected undifferentiated sarcoma based on imaging studies and tumor makers. Thus, we performed core needle biopsy, although open biopsy is often preferable to needle biopsy for the accurate diagnosis of liver tumors in children. If the result was ambiguous, we would have next performed open or laparoscopic biopsy in order to obtain an adequate volume of tumor tissue. According to the latest World Health Organization (WHO) criteria, PEComas are mesenchymal tumors composed of distinctive cells that show a focal association with blood vessel walls and which usually express melanocytic and smooth-muscle markers [14]. The key immunological markers for the precise diagnosis are α-SMA, HMB-45, and melan A [15]. In our case, some cells were positive for α-SMA and melan A, while approximately 50% of cells were positive for HMB-45. The percentage of HMB-45- and/or melan A-positive cells in each case was not documented or discussed in the relevant literature. In the figures presented by Folpe et al. HMB-45-positive cells and SMA-positive cells were focal (not the diffuse positive pattern), although the precise percentage of positive cells is not mentioned in the literature [3]. They indicated that some PEComa cases may be negative for melan A, highlighting the importance of careful evaluation or melanocytic differentiation, using both HMB-45 and melan A. Similarly, the ratio of α-SMA-positive cells was not clear. In the figures in a report by Nie et al. there seemed to be partial α-SMA positivity (not a diffusely positive pattern), indicating that SMA positivity may be variable in PEComa cases [16]. Folpe et al. also noted that occasional PEComas are actin-negative, raising caution that actin negativity does not exclude the diagnosis of PEComa [3]. We concluded that the tumor in our case is consistent with PEComa based on the histopathological findings along with the immunoreactivity with melanocytic markers and focal positivity for α-SMA.

The definitive criteria for distinguishing between benign and malignant lesions have not yet been established for PEComa. Folpe et al. proposed criteria for categorizing PEComas into three groups according to their pathological characteristics: benign, malignant and uncertain malignant potential [9]. The worrisome features in those criteria were as follows: (1) tumor size > 5 cm; (2) high nuclear grade; (3) hypercellularity; (4) mitotic rate of > 1/50 high-power fields; (5) necrosis; (6) infiltration into the surrounding normal parenchyma and (7) vascular invasion. Lesions with more than two of these features were considered malignant; the present case had all seven features. However, the fact that discrepancies between histological findings and clinical behavior have been observed in some PEComa patients should be noted. Partfitt et al. reported a case wherein hepatic PEComa with benign histological features presented with distant metastasis to multiple sites 9 years after surgery [5]. Apart from Folpe’s criteria, some immunohistochemical markers are noteworthy for allowing malignant PEComa to be distinguished from benign PEComa [17, 18]. Janks et al. suggests that immunohistochemical staining of CD117 may predict clinical behavior, on the basis that some studies have confirmed that tumor cells show diffuse cytoplasmic positivity for CD117 in benign AMLs, while the downregulation or loss of CD117 expression has been observed in a proportion of malignant AML cases [17]. We investigated the expression of CD117, and tumor cells were negative for CD117. The loss of CD117 expression in the tumor cells in our case supports its malignant potential.

^18^F-FDG PET/CT has also shown potential utility in the differentiation of malignant and benign PEComas. Previous reports have shown that all malignant PEComas (n = 4) demonstrated an intense FDG uptake in both the primary lesion and metastatic foci, with SUV_max_ values ranging from 3.19 to 72.2. However, 55 of 62 benign PEComas exhibited a low or negative FDG uptake on PET imaging, with SUV_max_ values lower than 2.0. [19]. The high uptake of FDG in our case also suggests malignancy. In contrast, a relatively high intensity on diffusion-weighted MRI is not necessarily consistent with malignancy. Case reports that describe the MRI findings in adults with malignant hepatic PEComa are limited in number (Table 1). The relationship between the diffusion-weighted MRI findings and malignant behavior were not clear in other studies [11, 16].

Surgical resection is believed to be the only curative treatment for PEComa. In the adult cases of malignant hepatic PEComa, lobectomy was most often performed, followed by trisegmentectomy, and partial hepatectomy (Table 1). One case underwent liver transplantation for recurrent disease [20]. The minimum degree of resection that is acceptable for hepatic PEComa has not been established, as the optimal surgical margins for hepatic PEComa are unclear. In our case, central bisegmentectomy was performed, as the tumor was located in the central portion of liver and the dorsal portion of S8 was preserved in consideration of the residual liver function. In volumetry the residual liver volume was estimated to be adequate even if the whole S8 was resected. However, we were concerned that the real residual liver function might be smaller than that estimated by volumetry considering the general condition, high level of inflammation, and hypoalbuminemia. Complete resection (R0) with a margin was possible, even preserving the dorsal portion of S8. Our patient subsequently presented with relapses at multiple sites. Whether or not the operative method influences the tendency for relapse is unclear. Nevertheless, it is important to prepare a surgical strategy to secure an adequate tumor margin according to the patient’s condition.

No effective medical treatment has been established for patients with advanced disease. Some authors have reported the efficacy of sirolimus, an oral mTOR inhibitor, for metastatic PEComa patients [21–23]. The activation of the mTOR pathway through the loss of the tuberous sclerosis complex 1 (TSC1)/TSC2 repressor complex seems to be a common pathogenic event in PEComas [24]. Thus, mTOR inhibitors may prove useful as a new therapeutic approach for PEComa [21]. In our case, genetic alteration in the TSC2 gene was identified in tumor cells, so an mTOR inhibitor was started after the second relapse. Even in adult cases of malignant hepatic PEComa, there is only one reported case that describes the use of an mTOR inhibitor as neoadjuvant therapy. The effectiveness of mTOR inhibitor treatment would affect the prognosis of patients with advanced malignant hepatic PEComa. Immunohistochemistry to detect p70S6K would be useful as a possible indicator of the efficacy of mTOR inhibitor treatment [6]. Unfortunately, we could not perform p70S6K immunostaining due to technical limitations.

Some researchers have proposed a short-term observation strategy [25, 26]. According to their strategy, when hepatic PEComa is suspected, FNAB combined with HMB-45 staining should be performed in all asymptomatic patients with lesions of < 5 cm in size who has shown no serological abnormalities. If the diagnosis is confirmed by FNAB and pathomorphology indicates a benign pattern, careful observation with serial imaging follow-up is recommended. Although this strategy seems to be practical, close attention is required, as some PEComas have the potential to grow very rapidly in a short period, as occurred in our case. Nguyen et al. suggested that the following features could be used for differentiation between benign and malignant hepatic PEComa: cytologic atypia, coagulative necrosis, larger tumor size (> 10 cm), CD117 negativity, and clinical evidence [27]. When hepatic PEComa is suspected in a pediatric patient—when possible—open or laparoscopic biopsy should be performed in order to obtain an adequate volume of tumor tissue. We recommend an immunohistochemical examination to detect CD117 and HMB-45. In case with a tumor of > 10 cm in size, a rapid growth pattern, or CD117 negativity, malignancy should be strongly suspected and surgical resection should be considered without delay. If the tumor is already inoperable or shows borderline resectability, mTOR inhibitor treatment should be considered—after confirming immunohistochemical positivity for p70S6K—as this may provide a chance to perform resection after the shrinkage of the tumor. Liver transplantation would be a final choice of treatment for cases of advanced disease without distant metastasis.

## Conclusion

Although extremely rare, malignant hepatic PEComa can develop in a child. It is important to achieve an accurate diagnosis of PEComa and to include it in the differential diagnosis of large liver masses, regardless of the patient’s age. Complete resection is the most important factor for ensuring long-term survival.

## Data Availability

The data that support the findings of this study are available from the corresponding author upon reasonable request.

## Abbreviations

PEComa: Perivascular epithelioid cell neoplasm
FNAB: Fine-needle aspiration biopsy
α-SMA: α-Smooth muscle antigen
HMB-45: Human melanin black-45
mTOR: Mammalian target of rapamycin
